# A promising approach in comparative research on care for the elderly

**DOI:** 10.1186/1741-7015-9-124

**Published:** 2011-11-18

**Authors:** Jouke van der Zee, Madelon Kroneman

**Affiliations:** 1 Department of International Health, Maastricht University, PO Box 616, 6200 MD Maastricht, The Netherlands; 2 NIVEL (Netherlands institute for Health Services Research), PO Box 1568, 3500 BN Utrecht, The Netherlands

## Abstract

Long-term care (LTC) in the form of care provided in nursing homes, homes for the aged and home care is considered an appropriate answer to the growing needs of the aging populations of the industrialized world. However, the provision of and expenditures on LTC vary considerably between these industrialized countries. Although one would expect LTC to be subject to many internationally comparative studies, including all European countries, this is not the case. A paper presented by Damiani *et al. *in *BMC Health Services Research *contains an internationally comparative model regarding the development of LTC in Europe (2003 to 2007). They achieve an intriguing compromise between depth and width in the sparsely populated domain of internationally comparative research on LTC by characterizing countries' LTC and interpreting the large north/south differences found. Their results also show that 'cash for care' schemes form a substantial alternative to traditional LTC provision. An additional time series analysis showed that many countries seem to be engaged in reorganizing the LTC sector. This study widens knowledge in a neglected area of health services research and should serve as a source of inspiration for further studies.

Please see related article: http://www.biomedcentral.com/1472-6963/11/316 [1]

## Introduction

Although the aging of the populations of the developed world, and the huge amounts of care needed to accommodate these aging populations, is considered to be one of the major policy problems for now and the near future [2], international comparative research including a broad number of countries is scarce. The main reason for this can be found in the complexity of the subject and the problems that are encountered concerning definitions and concepts in this area, which is found somewhere between health care, social services and informal care [3]. Substantial differences exist between European countries regarding all relevant aspects of care for the elderly, usually referred to as long-term care (LTC). Differences exist in the way countries organize care for the elderly; some countries rely heavily on family care, whereas others provide care in the form of publicly funded institutions and/or professional home care providers [4,5]. LTC expenditures vary accordingly [2,6] and there is a big north/south divide in the patterns of LTC in Europe [7,8]. As a result of the fuzzy boundaries between family care, social care and medical services, LTC research is notoriously complicated. This is also reflected in the validity, reliability and comparability of official statistics (see, for example, Van Mosseveld *et al. *[9]). One of the few research groups that tackled the international comparison of LTC is an Italian group, led by Damiani, who decided to use the available statistical data anyway (Damiani G *et al.* Patterns of long term care in 29 European countries: evidence from an exploratory study.)[1], showing that the use of international databases can lead to valuable results, and that sophisticated statistical methods can be meaningfully applied. The study of Damiani *et al. *contains all 27 European Union (EU)-member states plus Norway and Iceland (not Switzerland) and covers a recent five-year period (2003 to 2007). The authors use eight indicators derived from international databases, such as the Organisation for Economic Co-operation and Development (OECD) health data [6] and Eurostat data [10], about the health and functional status of the elderly (65 and older) population, the supply and utilization of LTC services and LTC expenditure.

The use of international databases brings about some additional limitations besides the validity of the data. Firstly, the databases are focused on utilization and expenditure of formal care. Data on family values concerning LTC and data on informal care are either lacking or, if available, often cover one year only and are thus not suitable for trend studies. Secondly, the scope of the database can be limited. For instance, the Eurostat database [10] only includes countries that are members of the EU and consequently data on Iceland, Norway and Switzerland are missing. As a result of the use of different definitions of LTC, data lacking in one database cannot simply be complemented from the other database. On the other hand, an advantage of these databases is that at least some efforts have been made to make the data comparable across countries.

Furthermore, the observation of a Danish poet, Piet Hein, who stated that experts seem to enjoy explaining why something cannot be done [11], appears to be valid here; experts seem to discourage the use of these databases (the OECD database even gives a message warning of the dangers of using data from different countries each time an international comparison is made). This may discourage the use of these databases, which, in our opinion, contain valuable information. In this respect we welcome the study of Damiani *et al.* (Damiani G *et al.* Patterns of long term care in 29 European countries: evidence from an exploratory study [1]). 

## Patterns and trends of LTC

The study by Damiani *et al. *consists of two parts: patterns and trends (Damiani G *et al. *Patterns of long term care in 29 European countries: evidence from an exploratory study.) [1]. In '*Patterns of care*', the authors suggest that the eight indicators derived from international databases can be reduced statistically to two 'factors'. Firstly, a factor labeled 'the alignment between elderly needs and old age related expenditures' indicates whether expenditure and demand are in balance. Secondly, a factor labeled 'supply of formal (mostly residential) care' is identified. With the help of the scores on both of these dimensions, four groups of countries can be distinguished, varying from one group scoring high on both factors (indicating a good balance between expenditure and need and a high formal supply of care) to a group scoring low on both factors, with the remaining two groups scoring low on one factor and high on the other. In '*Trends in LTC*', the authors show that countries differ in the way LTC develops over time. Some countries are stable; others have a linear development along one of the dimensions (factors) and some countries show a rather 'chaotic' time trend.

## What does this study add to the existing knowledge?

Until now, international studies were either statistical overviews of 27 to 35 countries with limited interpretation, or studies covering in depth a broad range of topics but for a much lower number of countries. A good example of the latter group is the Survey of Health Aging and Retirement (SHARE) study, covering 13 countries in its most recent version [12,13].

By combining the indicators into two dimensions, the study by Damiani *et al. *improved the interpretation of the existing international data. Additionally, by limiting themselves to a statistically defendable two-factor solution, the authors also avoided overstretching the possibilities that the relatively small number of 29 countries permits.

The dimensions presented also contain a new element, especially in the dimension they call 'the alignment between LTC expenditures and elderly needs'. The authors interpret this dimension as 'cash benefits' on the one hand (state and private pensions, 'cash for care' schemes), and the need (demand) for LTC in a country on the other, with the percentage of the population over 80 and self-assessed health status used as indicators of demand.

The study groups together countries like Luxemburg, Denmark, Italy, France and the UK, which have relatively low levels of supply of institutional LTC. This 'cash for care' dimension as a characteristic for dealing with the needs of the elderly population is a new concept. Also novel is the fact that former East Bloc countries still form a distinctive group, characterized by a weak alignment between LTC expenditures and the needs of the elderly, and a relatively high supply of formal care. If these countries provide LTC, they do it in the form of residential care, in 'beds'.

Finally, the trend analysis yields new insights, to a certain extent: trends could be established for each combination of the two dimensions. In some cases these were linear trends, with developments in one direction for one of the dimensions over the five years; in some countries, there existed a stable situation; in other countries, there were chaotic, highly volatile developments. The authors remark that some countries are in, or have apparently been in, a process of transformation - examples include Spain, Norway, Ireland, Italy, Portugal, Cyprus, Romania and Latvia. The trend analysis proves its relevance and should become a standard element of this type of international health care analysis. Changes occur rapidly, as was the case in the Netherlands. In the period between 2003 and 2007, LTC care in the Netherlands did not change much. In the years afterwards, however, the funding and organization of LTC changed considerably; even the 'cash for care' scheme is planned to be abolished in 2012 due to its popularity.

## Critical considerations

We value the attempt of Damiani *et al. *to make an international comparison of LTC (Damiani G *et al.* Patterns of long term care in 29 European countries: evidence from an exploratory study.)[1]. However, we have some critical comments that should be noted.

Firstly, we have some remarks concerning the concepts and definitions used in the paper. For instance, with regard to the social benefits indicator (in the paper interpreted as 'cash for care'), it is unclear whether these benefits also include capital based pensions as is customary in the UK, Denmark and the Netherlands and whether benefits in kind are also included. Moreover, it is unclear whether the self-assessed health indicator is a valid measure of 'health status or health need' or, rather, a culturally determined outlook on life. In international studies on 'happiness' [14], inhabitants of Mediterranean countries seem to be systematically less content than their Northern counterparts. Furthermore, the level of formal LTC (LTC beds in institutions other than hospitals) may be underestimated in countries like France, Romania, Malta and Ireland, where LTC beds form a substantial part of the supply of hospital beds (long stay beds, lits de longue séjour in France).

Secondly, we wondered whether there really is an 'alignment' between LTC expenditure and self-perceived health in a country or whether this is just a statistical association. There may be other issues that influence this relationship, such as supplier-induced demand [14]. Therefore we would recommend further research into the causality of these relationships.

Finally, an issue that has not been addressed in this study is the question of whether differences within countries could be more relevant than differences between countries; the authors themselves showed in an earlier paper the North/South differences in Italy [15], other papers have demonstrated East/West differences in Germany [16] and differences between Flanders and Wallonia in Belgium [17]. These differences within countries may be especially relevant in countries where health care is regionally organized and funded. Despite these ongoing questions, we cannot deny that the study by Damiani *et al. *forms a valuable contribution to internationally comparative LTC research and should inspire further research.

## Future directions

To that end, one of the intriguing elements of the Damiani paper is the attempt to interpret the international differences found by pointing to cultural dimensions following Hofstede's division [18], among others, into feminine (North Western) and masculine (South and Eastern) countries or into Roman Catholic (family-oriented) and Protestant (individual-oriented) countries. This latter dimension is also described as strong and weak family-value countries [12].

For the method used in the study by Damiani *et al*., time series are crucial. However, most time series data are only on expenditure and utilization of formal care. Thus valuable information, such as that provided by the Eurobarometer study on views and values concerning LTC [4]., are missing in the model This issue is acknowledged by the authors. Creating time series on values and views of the population of the EU on such an important issue as LTC would be extremely valuable in our opinion. This could be done by, for example, repeating the Eurobarometer study on a regular basis. Linking data on cultural and family values (and religion as background) and informal care to the expenditure and utilization that are analyzed in this study seems to be a promising way to reveal the mechanisms behind the patterns and trends in LTC in Europe.

## Conclusion

Damiani *et al. *achieved an intriguing compromise between depth and width in the sparsely populated domain of internationally comparative research on LTC. By reducing the set of eight indicators to two interpretable dimensions ('the alignment between old age-related LTC expenditures and needs of the elderly' and 'formal supply of LTC'), the authors increase the insight into LTC developments in Europe. Their use of time series analysis adds further insight into the field.

The interpretations the authors provide of the (already known) differences in the provision and use of LTC between the Protestant North and West and the Roman Catholic and/or Orthodox South (East) of Europe demand further in-depth research of the type that currently takes place on a smaller scale in a survey like the SHARE study [12]. Countries in Central and Eastern Europe follow a distinct path of development with innovations ('cash for care' schemes) that can be relevant for the rest of Europe, too.

To summarize, we think that the article of Damiani *et al. *(Damiani G *et al.* Patterns of long term care in 29 European countries: evidence from an exploratory study.) [1] contributes valuably to describing the dynamics of LTC in Europe, and widens knowledge in this neglected area of health services research in Europe.

## Competing interests

The authors declare that they have no competing interests.

## Authors' contributions

JvdZ wrote the first draft. Both authors contributed equally to further drafting and revision of the final manuscript, and gave approval for the final manuscript.

## Pre-publication history

The pre-publication history for this paper can be accessed here:

http://www.biomedcentral.com/1741-7015/9/124/prepub
